# Whole Genome Association Mapping of *Fusarium* Head Blight Resistance in European Winter Wheat (*Triticum aestivum* L.)

**DOI:** 10.1371/journal.pone.0057500

**Published:** 2013-02-22

**Authors:** Sonja Kollers, Bernd Rodemann, Jie Ling, Viktor Korzun, Erhard Ebmeyer, Odile Argillier, Maike Hinze, Jörg Plieske, Dagmar Kulosa, Martin W. Ganal, Marion S. Röder

**Affiliations:** 1 Leibniz Institute of Plant Genetics and Crop Plant Resesarch (IPK), Gatersleben, Germany; 2 Julius Kühn Institute (JKI), Braunschweig, Germany; 3 KWS LOCHOW GMBH, Bergen, Germany; 4 Syngenta Seeds S.A.S., Toulouse, France; 5 Syngenta Seeds GmbH, Bad Salzuflen, Germany; 6 TraitGenetics GmbH, Gatersleben, Germany; Kansas State University, United States of America

## Abstract

A total of 358 recent European winter wheat varieties plus 14 spring wheat varieties were evaluated for resistance to *Fusarium* head blight (FHB) caused by *Fusarium graminearum* and *Fusarium culmorum* in four separate environments. The FHB scores based on FHB incidence (Type I resistance)×FHB severity (Type II resistance) indicated a wide phenotypic variation of the varieties with BLUE (best linear unbiased estimation) values ranging from 0.07 to 33.67. Genotyping with 732 microsatellite markers resulted in 782 loci of which 620 were placed on the ITMI map. The resulting average marker distance of 6.8 cM allowed genome wide association mapping employing a mixed model. Though no clear population structure was discovered, a kinship matrix was used for stratification. A total of 794 significant (−log_10_(p)-value≥3.0) associations between SSR-loci and environment-specific FHB scores or BLUE values were detected, which included 323 SSR alleles. For FHB incidence and FHB severity a total of 861 and 877 individual marker-trait associations (MTA) were detected, respectively. Associations for both traits co-located with FHB score in most cases. Consistent associations detected in three or more environments were found on all chromosomes except chromosome 6B, and with the highest number of MTA on chromosome 5B. The dependence of the number of favourable and unfavourable alleles within a variety to the respective FHB scores indicated an additive effect of favourable and unfavourable alleles, i.e. genotypes with more favourable or less unfavourable alleles tended to show greater resistance to FHB. Assessment of a marker specific for the dwarfing gene *Rht-D1* resulted in strong effects. The results provide a prerequisite for designing genome wide breeding strategies for FHB resistance.

## Introduction

*Fusarium* head blight, caused by the fungal pathogens *Fusarium graminearum* and *Fusarium culmorum* is one of the most important fungal diseases of wheat and other cereals in the world. In addition to causing severe yield losses, *F. graminearum* is known to produce two important mycotoxins, deoxynivalenol (DON) and zearalenone, which can contaminate the diseased grains (http://www.wheatscab.psu.edu/PDF/Fusarium_Head_Blight_.pdf). The development and use of resistant cultivars is considered an important strategy for avoiding *Fusarium* infection. Resistance to *Fusarium* is quantitatively inherited. Therefore during the last decade a number of quantitative trait loci (QTL) mapping studies using molecular markers were conducted. The first mapped QTL originated from the Chinese spring wheat accession ‘Sumai 3’ or its derivatives [1]–[3]] and later efforts were undertaken to integrate these QTL into adapted germplasm [4]–[6]. This was followed by the identification and mapping of QTL for *Fusarium* resistance and mycotoxin accumulation in a number of bi-parental winter wheat populations [7]–[13], as well as efforts to introduce the novel QTL into elite background [14]. Meta-analysis of QTL locations summarized the mapping efforts and identified linked markers [15]–[17]. During the studies, the relationship between plant height and *Fusarium* head blight resistance became evident [18], and the effects of dwarfing genes *Rht-D1b* and *Rht-B1b* on susceptibility to *Fusarium* head blight were studied [19]–[21].

Genome-wide association studies (GWAS), originally developed in human genetics, are nowadays employed in numerous studies involving model and crop plants and are showing there much greater success with fewer resources than in human genetics (for review [22]). While the classical QTL-mapping is limited to bi-parental populations, GWAS identify the novel functional variation in a broad spectrum of varieties or accessions and thus are suitable for allele mining. Additionally, more meiotic events, that have taken place during evolution or cultivar development, are taken into account compared to bi-parental populations, resulting in an increased genetic resolution in dependence of the linkage disequilibrium (LD) of the species under investigation. In wheat, GWAS were reported for various traits including yield and agronomic traits [23]–[25], baking and milling quality [26]–[28], ear emergence [29], pre-harvest sprouting [30] and resistance to pathogens [31]–[34]. *Fusarium* head blight resistance was studied using an association genetics approach in barley [35] as well as in wheat germplasm [33].

The goal of this study was (1) to assess the resistance to *Fusarium* head blight in a set of 358 recent European winter wheat varieties and 14 spring wheat varieties as outgroup in four year/location combinations ( = environments) in the field, (2) to determine the marker-trait associations for resistance to *Fusarium* based on genome wide coverage using 732 SSR markers plus markers for candidate genes, and (3) to compare the obtained results with QTL reported in the literature.

## Results

### Phenotypic data analysis

**Figure 1:**
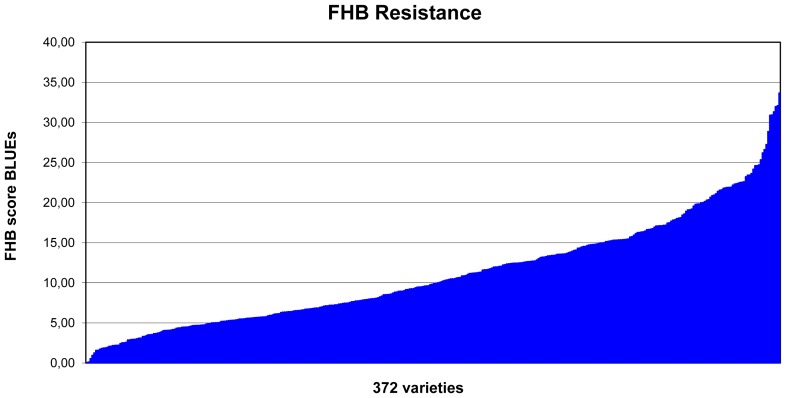
Phenotypic distribution of FHB score BLUEs in 372 varieties. The BLUEs of the FHB score (FHB incidence×FHB severity) were based on resistance tests in four environments. A low FHB score indicates high resistance.

FHB rating was based on four separate environments and BLUEs (best linear unbiased estimations) values were calculated across all environments (Table S1). For the FHB score, FHB incidence (Type I resistance)×FHB severity (Type II resistance) were taken into account. The Spearman rank correlation coefficients between BLUEs of FHB scores with BLUEs of FHB incidence and FHB severity were high with 0.991 and 0.958, respectively. Therefore the further analysis was focused on the FHB scores. FHB scores varied for the separate environments with means from 9.24 (2009.CEC) up to 11.83 (2010.BOD). The mean for BLUE values was 11.07. The BLUE values ranged from 0.07 (most resistant) to 33.67 (most susceptible) (Figure 1) and 27 varieties had a BLUE value smaller than 3.0 (Table S1). The two Swedish varieties ‘Kosack’ and ‘Stava’ were most resistant based on the BLUE values of 0.07 and 0.14 respectively.

Highly significant correlations between the environments indicated a good reproducibility of experiments (Table S2). The ANOVA analysis resulted in a high genotype effect (Table S3). Correlations of FHB-BLUE values with BLUE values for heading date and plant height evaluated in 8 different environments were negative and significant in both cases (Table S4). Thus later flowering varieties and taller varieties were more resistant.

### Molecular data analysis, linkage disequilibrium and population structure

The 372 varieties were genotyped with 732 microsatellite markers, of which 48 amplified more than one locus, resulting in a total of 782 loci spread across the 21 chromosomes. Heterozygote genotype calls were found for 2.6% of the data. With 4.8% missing data points, 276844 datapoints (95%) were available for the association analysis. For the construction of a genetic map using the ITMI (International Triticeae Mapping Initiative) recombinant inbred mapping population, a LOD score of 3.0 was taken as the threshold to declare linkage. A total of 620 markers were placed on this map, with 19 SSR markers mapping to more than one position in the genome, resulting in a map of 4470 cM length. The remaining markers could not be mapped onto this population due to lack of data or polymorphism. Altogether, this resulted in an average marker distance of 7.2 cM (ranging from 4.6 cM on chromosome 4B to 10.2 cM on chromosome 6A). The genome was evenly covered, with the D-genome being slightly overrepresented (38.5% of the markers) and homoeologous group 6 being slightly underrepresented (9.8% of markers).

**Figure 2:**
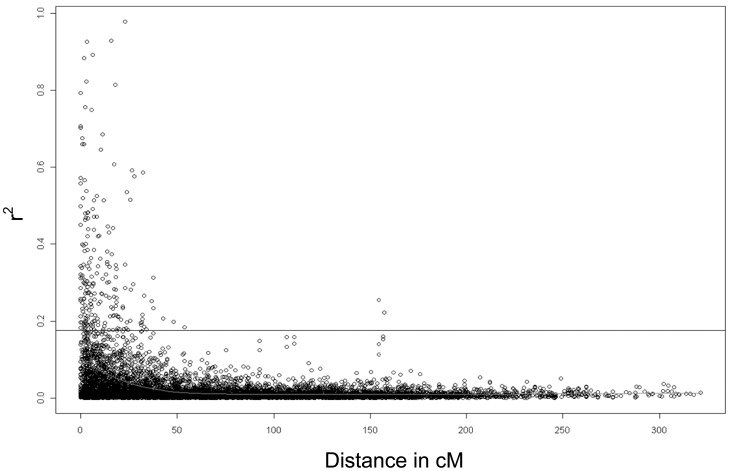
Linkage disequilibrium represented as r^2^ of marker pairs versus genetic distance in centiMorgan over all chromosomes. The horizontal line marks the threshold above which LD is likely due to genetic linkage.

Linkage disequilibrium (LD) is described with the parameter r^2^. Of the 8146 marker pairs, which returned r^2^ values, 62.9% (5121) showed statistically significant (p<0.05) LD as determined via 10,000 permutations. The average r^2^ for loci on different chromosomes (unlinked loci) was 0.031. R^2^ was in general low, being higher between neighbouring marker pairs (0.03 on chromosome 6A to 0.14 on chromosome 4B) than between all marker pairs on the same chromosome (0.02 on chromosomes 1B, 2D, 3A, 3B, 3D, 5D, 6A, 6D, 7A and 7D to 0.08 on chromosome 4D) and reached its lowest value for marker distances above 50 cM with 0.01 between all marker pairs on the same chromosome. For distances below 10 cM it was slightly higher (0.09 for all marker pairs on the same chromosome and 0.11 for neighbouring marker pairs) than for distances between 10 to 20 cM (0.05 for all marker pairs on the same chromosome and 0.03 between neighbouring marker pairs). The critical value for LD was 0.176. Above this value LD was considered to be due to genetic linkage. Only 207 marker pairs were in LD because of genetic linkage since their r^2^ was higher than 0.176 (Figure 2).

**Figure 3:**
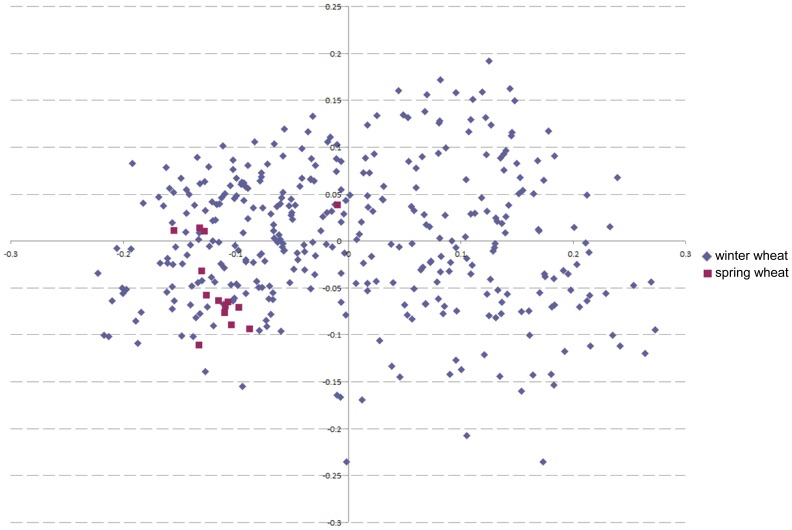
Analysis of population structure. Principal coordinate analysis of 358 winter varieties and 14 spring wheat varieties was conducted with 155 SSR markers.

Population structure was assessed via principal coordinate analysis employing Rogers' modified distance (Figure 3). No distinct substructure was observed. Even distinguishing the varieties according to breeder or growth type (spring or winter) did not result in distinctive groups.

### Marker-trait associations

**Figure 4:**
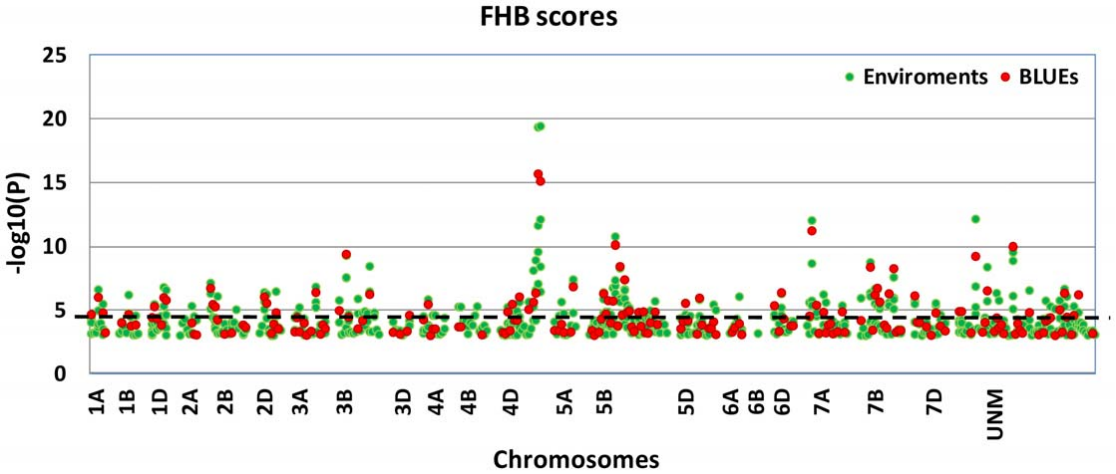
Manhattan plots of marker-trait associations for FHB resistance. The plot represents the individual significant −log_10_(p)>3.0 marker-trait associations of four environments plus BLUEs sorted according to their chromosomal location. The dotted line indicates the threshold of −log_10_(p) = 4.82 for Bonferoni correction. All markers which were not associated or associated with a −log_10_(p) below 3.0 were set to 0. Green dots represent the MTA of a single environment, red dots represent the MTA of a BLUE value.

Associations were calculated individually for each environment and for the BLUEs. Using the software package Genstat 14^th^ edition a total of 794 individual significant (−log_10_(p)-value≥3.0) associations between SSR-loci and FHB scores were detected using a mixed linear model with kinship matrix (Fig. 4; Table S5). For the components FHB incidence a total of 861 marker-trait associations (MTA) and for FHB severity a total of 877 associations were found. In most cases the MTA for FHB severity and FHB incidence co-located with each other and also co-located with FHB score. Only few loci (WMC612, GWM285, GWM376b, GWM803) on chromosome 3B were specific for severity.

For FHB score, a total of 157 mapped markers showed MTA; often two or several different alleles of a marker were significant and most MTA were detected in several environments (Fig S1; Table S5). All chromosomes showed MTA, though for chromosome 6B only one individual MTA was detected. The highest number of MTA was detected for chromosome 5B with two linkage blocks of neighbouring markers on the reference map (GWM843, GWM213, GWM335, GWM1108 and GWM1475, GWM777, WMC75, GWM408). Similar linkage blocks of four markers were also seen on chromosome 3A (BARC294, BARC321, BARC57, BARC129) and chromosome 4D (BARC98, WMC437, GWM819, GDM129).

Since microsatellites are multi-allelic markers, different alleles may show different kinds of effects. A negative additive effect, which means increasing resistance, was detected for 237 MTA for FHB score, while 557 MTA showed positive additive effects, meaning decreasing resistance. Both kinds of effects were often detected for the same marker, depending on the respective alleles.

**Figure 5:**
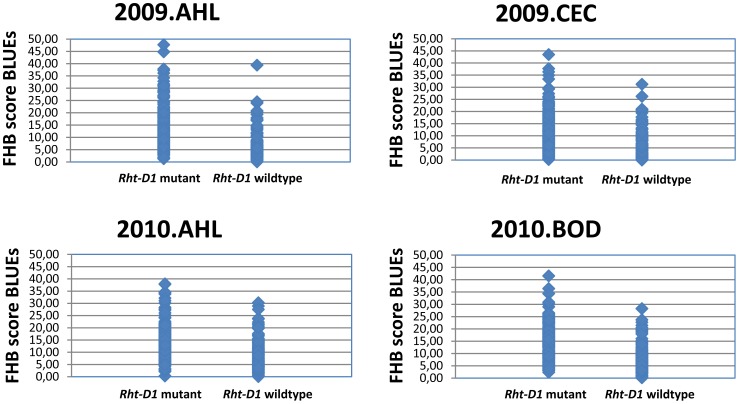
Allelic effects of dwarfing gene *Rht-D1* in a population of 372 European wheat varieties. Varieties carrying the mutant allele *Rht-D1b* (dwarfing type) showed an increased FHB score resulting in decreased resistance in four different environments.

The marker data of two known genes, previously reported to have an influence on *Fusarium* resistance, were applied in this association analysis. For the dwarfing gene *Rht-D1* (formerly called *Rht2*) on chromosome 4D significant associations were observed in all environments (Table S5) for FHB score, severity and incidence. In all cases the mutant allele (dwarfing type) had positive additive effects, meaning decreasing resistance, while the wild type allele showed negative additive effects, meaning increasing resistance (Fig. 5). For the photoperiod sensitivity gene *Ppd-D1* (formerly *Ppd1*) on chromosome arm 2DS, the photoperiod insensitive *Ppd-D1a* mutant had a positive additive effect, increasing FHB severity and decreasing resistance, as compared to the photoperiod sensitive wild type with negative additive effect, increasing resistance for FHB severity (Table S5).

### Additive effects of favourable and unfavourable alleles

**Figure 6:**
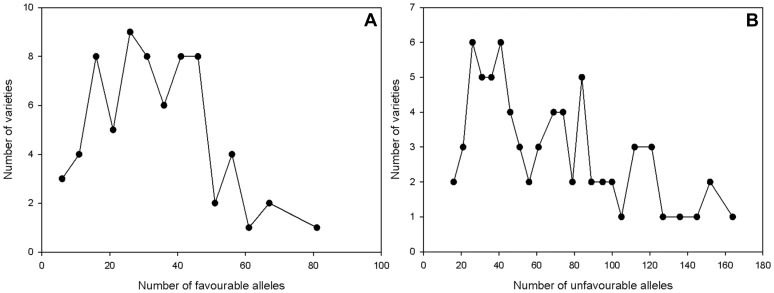
Frequency of (A) favourable FHB alleles and (B) unfavourable FHB alleles in individual varieties. Most varieties carried between 20 to 40 alleles increasing or decreasing the resistance to FHB.

In total 114 alleles with favourable (resistance increasing) and 209 alleles with unfavourable (resistance decreasing) effects were detected for FHB score (Table S5). The number of favourable alleles per variety ranged from 6 to 81, while the number of unfavourable alleles per variety ranged from 16 to 167 with most varieties carrying 20 to 40 alleles of each kind (Fig 6). The correlation coefficient of the number of favourable alleles in a variety with its FHB score BLUE values was −0.730 and the correlation coefficient for unfavourable alleles with FHB score BLUEs was 0.703.

**Figure 7:**
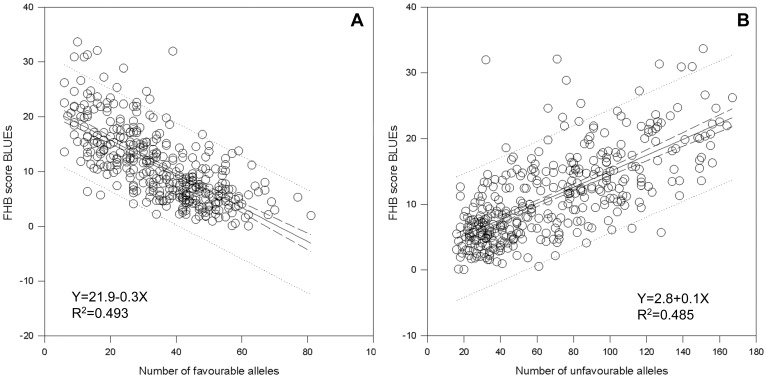
Effect of (A) favourable and (B) unfavourable FHB alleles. Linear regression resulted in a relationship of FHB-BLUEs score and number of favourable and number of unfavourable alleles in 372 wheat varieties.

Linear regression showed a dependence of the FHB score BLUEs on the number of favourable alleles per variety with r^2^ = 0.493 and Y = 21.9–0.31 X. For unfavourable alleles, the same type of analysis resulted in r^2^ = 0.485 and Y = 2.8+0.12 X. This indicated a certain additive effects for favourable as well as unfavourable FHB-alleles (Fig. 7).

The two most resistant varieties ‘Stava’ and ‘Kosack’ carried 57 and 62 favourable and 17 and 20 unfavourable alleles, respectively, confirming, that the numbers of favourable and unfavourable alleles in a variety influence the degree of resistance to FHB.

## Discussion

### Population structure

It is known that population structure can influence association results and the lack of appropriate correction for population structure can lead to spurious associations [36], [37]. In this context, it can be regarded as beneficial that our association population did not show any population structure, even though we conducted a stratification using a kinship matrix. Another study detected the presence of population structure in European elite wheat varieties by using SSR and DArT markers [38]. However, in contrast to our study this study included a significant portion of UK varieties, which were the first group to separate from the German and French varieties. The reported further separation in two French groups and one German group of plant material [38] was not observed in our material, which also contained some varieties from other countries like Denmark, Sweden and Poland.

### Comparison to published FHB QTL

Genotyping of 732 microsatellite markers allowed the genome wide association mapping of FHB QTL in European winter wheat in this study. An earlier association genetics study [33] used European winter wheat germplasm. However the analysis contained only 115 SSR markers, which did not provide a complete genome coverage. The comparison of our results to published FHB QTL and meta-QTL is often rendered difficult due to the usage of different markers and maps. To some extent this was solved by the use of a very large number of SSR markers, so that many markers employed in previous studies were also used here. Furthermore, as reference we used the wheat consensus map [39] as well as the ITMI-map containing all GWM-markers [40].

#### Chromosome Group 1

On chromosome 1A, three loci were detected in at least three environments and BLUEs. The MTA for GWM357 on chromosome arm 1AL coincided with a meta-QTL described for the interval GWM357-BARC119 [17]. The other two MTA (GWM164 and GWM1097) were located on chromosome arm 1AS, for which also several meta-QTL were reported [16]. While several authors reported FHB-QTL on chromosome arm 1BS [15], [16], this locus was not found in our study. A MTA associated with WMC626 in the centromeric region of chromosome 1B was detected, which maps closely to the meta-QTL described for the interval BARC137-WMC320 [17]. The MTA associated with WMC631 in three environments including BLUEs mapped only 1.1 cM from GWM124 for which a meta-QTL was described [17]. Also the MTA for GWM3166 and WMC728 at the end of chromosome arm 1BL overlapped with described meta-QTL for the interval BARC80-GWM259 [17], [16], [11].

For chromosome 1D, CFD72 and WMC336 were reported as significant in European material [33]. Though both markers were also included in our association study, we did not detect any significant associations with these markers, but marker CFD15, which maps closely to WMC336, was significant in all environments plus BLUEs. Overall six loci were significant for chromosome 1D in at least three environments including BLUEs (Fig S1), while in the literature chromosome 1D is only sparsely covered with QTL or meta-QTL. The MTA of BARC152 may coincide with a meta-QTL which extends north of GWM106 [16], [15].

#### Chromosome group 2

As dominant feature, on the end of chromosome 2BS three closely linked markers (WMC154, WMC257, GWM1128) were significant in at least three environments and BLUEs. While no meta-QTL for this region were described, marker BARC45, mapping in a similar region, was found significant in an association analysis study [33]. In a more proximal region of chromosome arm 2BS, the MTA detected by markers GWM374, GWM972 and WMC344 coincided with a meta-QTL described for the interval WMC272-WMC171 in a similar genomic region [17]. On chromosome 2D, marker CFD116 detected resistance increasing as well as resistance decreasing effects with various alleles in four environments including BLUEs, however, no matching meta-QTL was found in the literature. The MTA discovered for CFD233 on chromosome arm 2D co-localizes with the meta-QTL described for interval GWM608-WMC261 [17] and the MTA linked to GWM1419 on the same chromosome arm coincides with the genomic region of meta-QTL at neighbouring marker GWM157.

#### Chromosome group 3

The MTA discovered on chromosome arm 3AS by four linked markers (BARC294, BARC321, BARC57, BARC12) co-located with a meta-QTL for resistance to spread linked to BARC12 [17]. A further proximal meta-QTL on chromosome arm 3AS, linked to GWM2 [16], [15], may coincide with the MTA detected with GWM1507 and GWM4018 in our study, while for the MTA discovered on chromosome arm 3AL in several environments (markers CFA2193, GWM1757, GWM1229) no matches in literature were found.

On chromosome arm 3BS, the well-known QTL derived from ‘Sumai 3’ is located [1]. Since our plant material mainly consisted of European winter wheat varieties, we did not expect the presence of this QTL in our study. In the respective genomic region only one MTA was detected for GWM493 and MTA for three environments for BARC75, while the highly significant marker WMC808 is located further proximal. In the central region of chromosome 3BS we found several markers (WMC612, GWM285, GWM376) significant specifically for FHB severity. This region coincides with described meta-QTL for severity in a similar genomic region linked with GWM285 and GWM376 [15]–[17]. For two linkage blocks with significant markers in multiple environments on chromosome 3BL (GWM3144, GWM1015, GWM4155 and GWM938, WMC291) no matching meta-QTL were described. Also for the MTA with marker BARC284 at the end of chromosome 3DS no co-locating QTL were found in the literature.

#### Chromosome group 4

The MTA detected by marker CFD71 on chromosome arm 4AS in several environments mapped closely to marker GWM165, which is linked to a major gene controlling resistance to Fusarium head blight type I resistance [41]. We detected significant associations for FHB score, FHB incidence as well as FHB severity in this region. In the region of WMC262 on chromosome arm 4AL, a QTL for spread of infection within the head was described [42]. The other MTA on chromosome 4A, which were significant in several environments and detected by markers GWM445 and CFA2256, did not have any matching meta-QTL. Also for the MTA detected by markers GWM891, BARC163 and WMC47 on chromosome 4B, no co-locating meta-QTL were described.

Chromosome 4D is characterized by a highly significant linkage block of four markers (BARC98, WMC473, GWM819, GDM129), which are located in the neighbourhood of *Rht-D1*. Three of these markers were in highly significant LD with *Rht-D1* (Fig S2). We did not include *Rht-D1* in our reference map, because no mapping data were available, however mapping positions are indicated elsewhere [16], [15]. Additional markers on chromosome arm 4DL were significant in several environments (GWM4346, GWM165b, WMC331, GWM4001). For marker WMC331 matching QTL were described in spring wheat populations [42], [43].

#### Chromosome group 5

A QTL linked to markers GWM129 and GWM304 was described for ‘Sumai 3’ derived material [2] on chromosome arm 5AS. This region also carried a very prominent meta-QTL, which was discovered in many mapping populations [16], [15]. We found a QTL linked to GWM415 and to BARC117 on chromosome 5A in one environment each, while markers WMC805 and WMC705, which were detected in multiple environments, were located further distal in our reference map. The MTA detected by marker BARC4 in several environments matches with a QTL from cultivar ‘Frontana’, mapped to the interval GWM129-BARC197 on chromosome 5A [44].

Chromosome 5B was the chromosome, where the highest number of individual MTA was detected, and it was characterized by two highly significant linkage blocks of four markers each (GWM843, GWM213, GWM335, GWM1108 and GWM1475, GWM777, WMC75, GWM408). While several QTL regions were described for chromosome arm 5BL [7], we could not verify a match with the described linkage blocks in our study based on the available marker coverage. Another linkage block at the end of chromosome arm 5BL in our study, involving markers BARC59, GWM1016, GWM4209, was located in a similar region like the QTL described for the marker interval GWM1246-PSR145 [7]. There was no counterpart for the MTA detected on chromosome 5D in the literature.

#### Chromosome group 6

Homoeologous group 6 was the most sparsely covered group with significant associations in our study. While for chromosome 6B a ‘Sumai 3’ derived QTL [1] and very prominent meta-QTL were described [15], [16], we detected a single MTA for this chromosome which was located more distal than the described meta-QTL. On chromosome 6A a major QTL was reported for the wheat varieties ‘Apache’ and ‘Dream’ [8], [45]. Marker BARC107, for which we detected several significant MTA, mapped closely to the indicated QTL intervals, but apparently was not covered by them. Multiple MTA were also discovered for marker GWM427 on chromosome arm 6AL; in a similar region a QTL was reported for the ‘Arina’/’Forno’ population spanning the interval GWM169-PSR966 [7]. On chromosome 6D, four markers detected MTA in at least three environments including BLUEs (GWM469, BARC273, GWM1401, GWM1103), however, none of them covered the QTL linked to GDM14 derived from variety ‘Arina’ [7].

#### Chromosome group 7

On the end of chromosome 7AS, six linked markers (WMC479, GWM681, GWM735, WMC168, GWM3064, CFA2049) showed MTA in multiple environments. A QTL for chromosome arm 7AS was described for cultivar ‘Frontana’ [46], though the available mapping information is quite imprecise. Another association analysis study [33] found significant associations for marker WMC596 on chromosome 7A, which is in agreement with our results. Marker BARC49 on chromosome 7A, with MTA for three environments including BLUEs, is the neighbouring marker to GWM276, for which meta-QTL were reported [16], [15]. Also the location of multiple MTA, detected by marker BARC182 at the end of chromosome 7BL, matches the region of described meta-QTL [16], [15]. Marker GWM400 at chromosome arm 7BS is located in the QTL-region described for variety ‘Dream’ [8] as well as several meta-QTL [16], [15]. Marker BARC267 on chromosome 7B is closely linked to GWM46, for which meta-QTL are described [16], however, for five further markers with multiple MTA in the central region of chromosome 7B (WMC 662, GWM808, BARC176, BARC278, WMC517) no matching QTL were reported. For several associations on chromosome 7D, no corresponding QTL were found in the literature.

### Effects of plant height and flowering time

We found strong effects for the dwarfing gene *Rht-D1*, with the mutant allele decreasing the resistance. A general correlation was detected between plant height and FHB resistance (Table S4). The same effect was described for nearly isogenic lines of *Rht-B1* and *Rht-D1* alleles in the background of ‘Mercia’ and ‘Maris Huntsman’ [19]. However, the authors pointed out, that the negative effects of short plant height on FHB susceptibility can be counteracted by a more resistant genetic background. Coincident meta-QTL between plant height and FHB resistance were reported [18]. It was argued [20], that the effect of *Rht-D1b* may not only be an indirect effect, caused by a changed microenvironment due to shorter plants, but that there may be a direct effect of *Rht-D1b* on the susceptibility to FHB, since *Rht-D1b* affects type I resistance (incidence) rather than the type II resistance (spread of pathogen). However, in our dataset we detected significant MTA of *Rht-D1* for FHB score, as well as FHB incidence and FHB severity. We did not find any effects for *Rht-B1* (former *Rht1*) on chromosome 4BS. The reason may be, that this dwarfing gene was present in only few varieties. The two most resistant varieties ‘Stava’ and ‘Kosack’ carried the wild type alleles for *Rht-D1* as well as for *Rht-B1*. In terms of plant height they ranked on position 227 and position 112 out of 372 varieties, which represented the medium range of plant height. This indicates that besides the absolute plant height other factors influenced by *Rht-D1* may affect the resistance behaviour.

Early flowering varieties and varieties carrying the photoperiod insensitive *Ppd-D1a* mutant showed decreased resistance. Since the inoculation process was repeated three times during the flowering of the tested varieties, we assume that this procedure compensated for variation in the effectiveness of inoculation caused by variation in the flowering time of the varieties. The spring varieties flowered on average two days earlier than the winter varieties, however in both types of varieties there were early and later varieties. While early flowering varieties may have experienced more than one inoculation event, also the weather conditions at the time of flowering at the respective trial sites play a role and can influence the spread of disease. The high correlations of FHB score between independent trial sites indicated that the procedure used is reliable and reproducible concerning the ranking of varieties.

A significant marker-trait association was detected for the *Ppd-D1* alleles based on FHB severity. Opposite effects were reported [44], [10] where early plants were more resistant. While these reports relate to bi-parental mapping populations, the observation in our data set may represent a population-inherent effect. The *Ppd-D1a* mutant allele was mainly observed in French varieties, since it has most impact in southern latitudes of Europe. On the other hand this germplasm often had higher susceptibility to FHB.

### Implications for plant breeding

The use of genome wide marker association provides a comprehensive analysis of the FHB resistance QTL present in Central European germplasm. As phenotypic data we used a combination of type I (incidence) and type II (severity; spread) resistance, which provided a good estimation of practical field resistance. The overall resistance of a variety seems to be controlled by a relatively high number of loci with resistance decreasing or increasing effects. Nevertheless, several of the tested varieties showed a good general resistance to FHB in various environments. The observed additive effects of favourable and unfavourable alleles encourage strategies of QTL-pyramiding. The avoidance of susceptibility loci may also increase the general FHB resistance. Due to the high number of loci involved, breeding for quantitative FHB resistance will require strategies of genomic selection [47], [48]. The advent of novel genotyping and sequencing techniques will provide the necessary genomic tools for such attempts [49]–[51]. At the present state of art, genome wide microsatellite data were useful in the discovery of marker-trait associations. This type of marker is also very suitable to follow the inheritance patterns of individual QTL incorporated into backcross breeding programs due to their multi-allelic nature. It will be interesting in future studies to compare the marker-trait associations of the multi-allelic microsatellite markers with those of bi-allelic SNPs.

## Material and Methods

### Plant material, field trials and disease evaluation

A total of 358 European winter wheat varieties and 14 spring wheat varieties were evaluated in this study (Table S6). Spring wheat varieties and winter wheat varieties were sown at the same time. All varieties were grown in two locations in Germany during the season 2008/2009 (2009.AHL (Ahlum), 2009.CEC (Cecilienkoog)) and in two locations in the season 2009/2010 (2010.AHL, 2010.BOD (Halle-Bodenwerder)) with three complete replicates per location.

Spray inoculations were performed with 50000 spores per ml of *Fusarium graminearum* and *Fusarium culmorum* isolates (1/3 F.g.∶2/3 F.c.) using a water volume of 600 L/ha. Each wheat variety was inoculated on three dates at intervals of 3 days with the spore suspension. It was started at the beginning of flowering (at stage BBCH 61) and finished at the end of flowering (at stage BBCH 65/69). Since not all varieties flowered simultaneously, some experimental variation between early and later flowering varieties may exist. The experiment was designed so that each variety received at least one inoculation during its flowering time. To determine the variety resistance against *Fusarium* head blight (FHB) the parameters incidence and severity were recorded. FHB incidence (Type I resistance) was visually rated as the percentage of infected spikes from 50 observed spikes per plot. Severity (Type II resistance) was rated as the percentage of infected area per spike of the infected spikes [52]. Three assessments were performed in each trial site 20, 28 and 33 days after the first inoculation, except for 2009.CEC, where only two assessments could be performed (28 and 33 days after inoculation) due to low disease pressure. Three replications were conducted per genotype and environment. FHB incidence and FHB severity were each calculated as means of three replications and three assessments per genotype and environment. FHB score was calculated as (FHB incidence x FHB severity)/100 separate for each environment.

The varieties were also evaluated for heading date and plant height in a companion study in eight different locations over the same two seasons.

### Molecular data analysis

DNA extraction and PCR were performed as described by [53]. The 372 varieties were genotyped with 732 microsatellite markers following standard protocols on capillary sequencing machines. Markers covered all 21 chromosomes and map positions on the ITMI mapping population (International Triticeae Mapping Initiative) were determined with the programme MAPMAKER v 3.0 [54] using the Kosambi mapping function [55] with a LOD score of 3.0 as the threshold for linkage. The mapping data were retrieved from the GrainGenes 2.0 database (http://wheat.pw.usda.gov/GG2/index.shtml) and from own formerly performed mapping endeavours [40]. Mapping was performed by only including the markers that had been used in the association analysis. Since the D-genome is underrepresented in many studies, more markers for the D-genome were included in the association analysis than for the A- and B-genomes. All the varieties were additionally genotyped for candidate genes *Rht-B1* and *Rht-D1* [56] and the *Ppd-D1a* allele of the photoperiod response locus *Ppd-D1* [57].

Population structure was inferred with a principal coordinate analysis based on modified Rogers’ distance [58] which was calculated with a subset of 155 loci. Principal coordinate analysis was performed in R using the function cmdscale. The 155 markers were chosen to be distributed across the genome and selected for the lowest number of missing data points, no null-alleles and lowest number of heterozygotes. A kinship matrix was calculated using the software SPAGeDi [59] based on the aforementioned 155 markers. Negative values were set to 0.

Linkage disequilibrium was measured with the parameter r^2^ calculated with the programme TASSEL v. 2.1 [60]. P-values for the pair wise significance of LD were derived with 10,000 permutations. LD was calculated for all marker pairs and subsequently analysed separately for each chromosome for all possible marker pairs and for pairs of neighbouring loci and for unlinked loci (on different chromosomes). LD decay was examined by graphs of pair wise distances (cM) versus r^2^. The LD decay was estimated at the point where a second degree Loess curve, which was calculated with the function loess.smooth in R, intersects the threshold of the critical LD. Critical LD was evaluated following [26]. The r^2^ values for all available marker pairs on different chromosomes were square root transformed and the 95^th^ percentile was chosen as the threshold above which LD is likely due to genetic linkage.

### Statistical analysis of phenotypic data

Each year-location combination was considered as an environment in our study. For each environment, the FHB incidence value (Type I resistance), the FHB severity value (Type II resistance) and FHB score value (incidence*severity)/100 were calculated separately for every genotype. For each genotype, the arithmetic mean for phenotypic observations in three replications and three assessments per genotype were generated and results were used as the phenotypic data for association analysis. In addition, best linear unbiased estimators (BLUEs) across all four environments were estimated by assuming fixed genotypic effects. Since the datasets for all environments were complete, the BLUEs, in fact, equalled the arithmetic means across environments. The calculation was performed by GenStat 14^th^ edition [VSN International, Hemel Hempstead, Hertfordshire, UK] using the “Mixed Models REML” module and the “Linear Mixed Models” function.

For estimating correlations, Spearman rank correlations were calculated with the software package SigmaPlot 11.0.

### Association mapping

For the calculation of genotype-phenotype associations, microsatellite data were converted into a bi-allelic data format, resembling SNP data. Each allele was considered as a single marker and genotypes were coded as ‘A’, when the variety possessed the allele and ‘C’ otherwise. A minor allele frequency threshold of 3% (equalling 11 varieties) was set and alleles with a lower frequency were excluded from the analysis. After filtering process, 3176 alleles remained and were employed for the association mapping approach. The FHB incidence value, FHB severity value and FHB scores value were used as trait values. Marker-trait associations were calculated separately for the four environments and the BLUEs across all environments. Calculations were performed with the software packages GenStat 14^th^ edition. In GenStat, the “QTL analysis” module and the “Single trait association analysis” function was utilized, and the kinship matrix was chosen as the relationship model.

## Supporting Information

Figure S1 **Chromosomal location of marker-trait associations.**
(PDF)

Figure S2 **Analysis of linkage disequilibrium (LD) in the region of** ***Rht-D1*** **on chromosome 4D.**
(PDF)

Table S1 Fusarium head blight scores of 372 varieties. FHB scores (FHB incidence×FHB severity) were assessed in four environments. The BLUEs were calculated across all environments using restricted maximum likelihood (REML). A low FHB score relates to high resistance, while a high FHB score relates to high susceptibility to FHB.
(XLSX)

Table S2 Spearman rank order correlations of FHB score of 372 varieties among four environments and BLUEs. *** P<0.001.
(DOCX)

Table S3 Analysis of variance of FHB score in 372 varieties in four environments.
(DOCX)

Table S4 Spearman rank order correlations of FHB-BLUEs of 372 varieties with HD-BLUEs (heading date) and PH-BLUEs (plant height). P<0.001.
(DOCX)

Table S5 List of significant (−log_10_(p)>3.0) marker-trait associations. Columns G to J describe the additive effect for each environment. A positive additive effect means that the resistance to FHB is decreased, a negative additive effect means that the resistance to FHB is increased.
(XLSX)

Table S6 Information about varieties.
(XLSX)
