# Supplementary material for: Whole Genome Association Mapping of Fusarium Head Blight Resistance in European Winter Wheat (Triticum aestivum L.)
Source: PLoS One. 2013 Feb 22;8(2):e57500. doi: 10.1371/journal.pone.0057500 (PMC3579808; doi:10.1371/journal.pone.0057500)
Supplement: Figure S1 — Chromosomal location of marker-trait associations. (PDF) [file pone.0057500.s001.pdf]

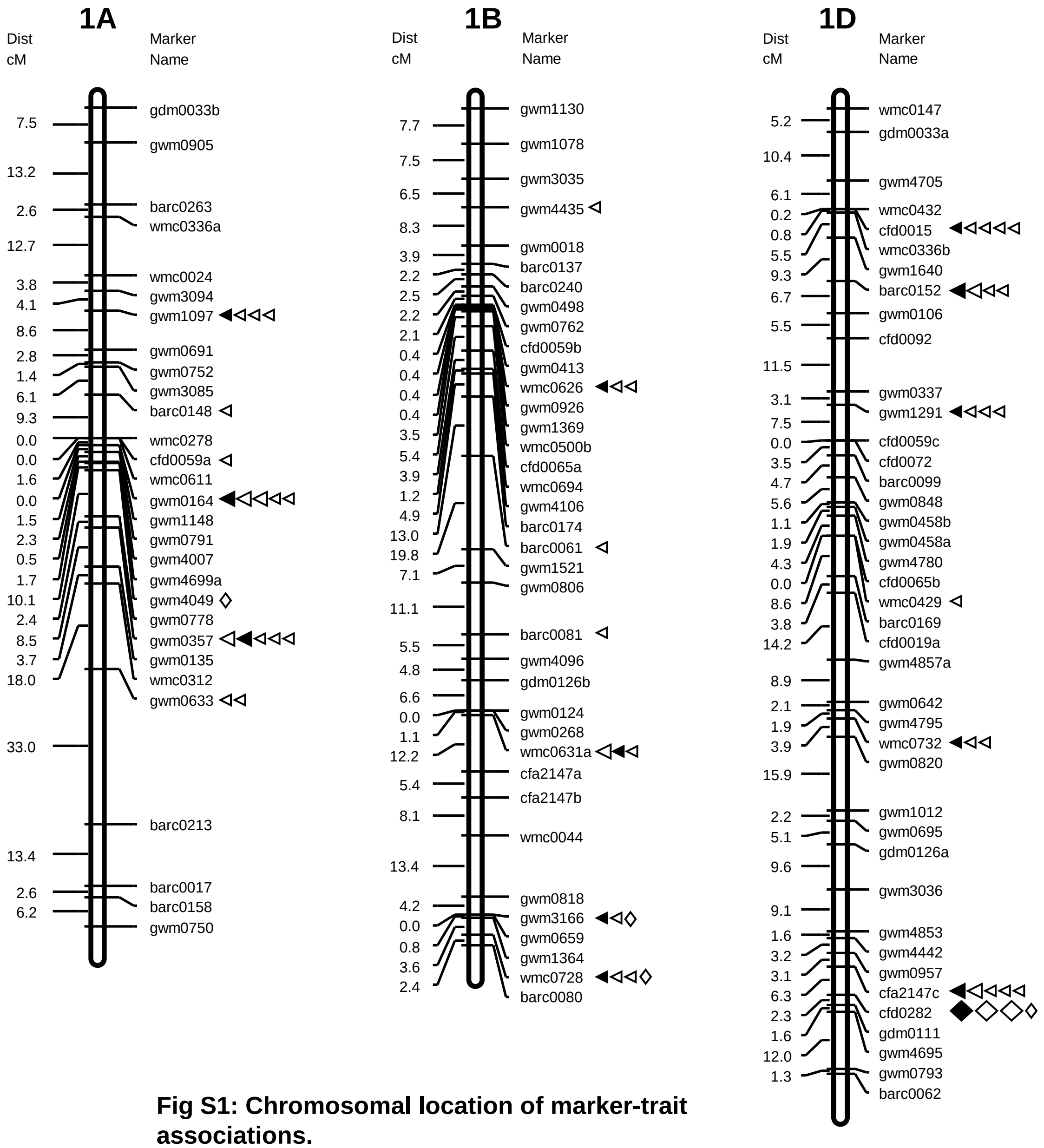

Fig S1: Chromosomal location of marker-trait associations.

◃ Resistance decreasing effect, single environment, -log10(p)-value > 3.0
◁ Resistance decreasing effect, single environment, -log10(p)-value > 4.82
◂ Resistance decreasing effect, BLUEs-value, -log10(p)-value > 3.0
◀ Resistance decreasing effect, BLUEs-value, -log10(p)-value > 4.82

◊ Resistance increasing effect, single environment, -log10(p)-value > 3.0
◇ Resistance increasing effect, single environment, -log10(p)-value > 4.82
⬧ Resistance increasing effect, BLUEs-value, -log10(p)-value > 3.0
◆ Resistance increasing effect, BLUEs-value, -log10(p)-value > 4.82

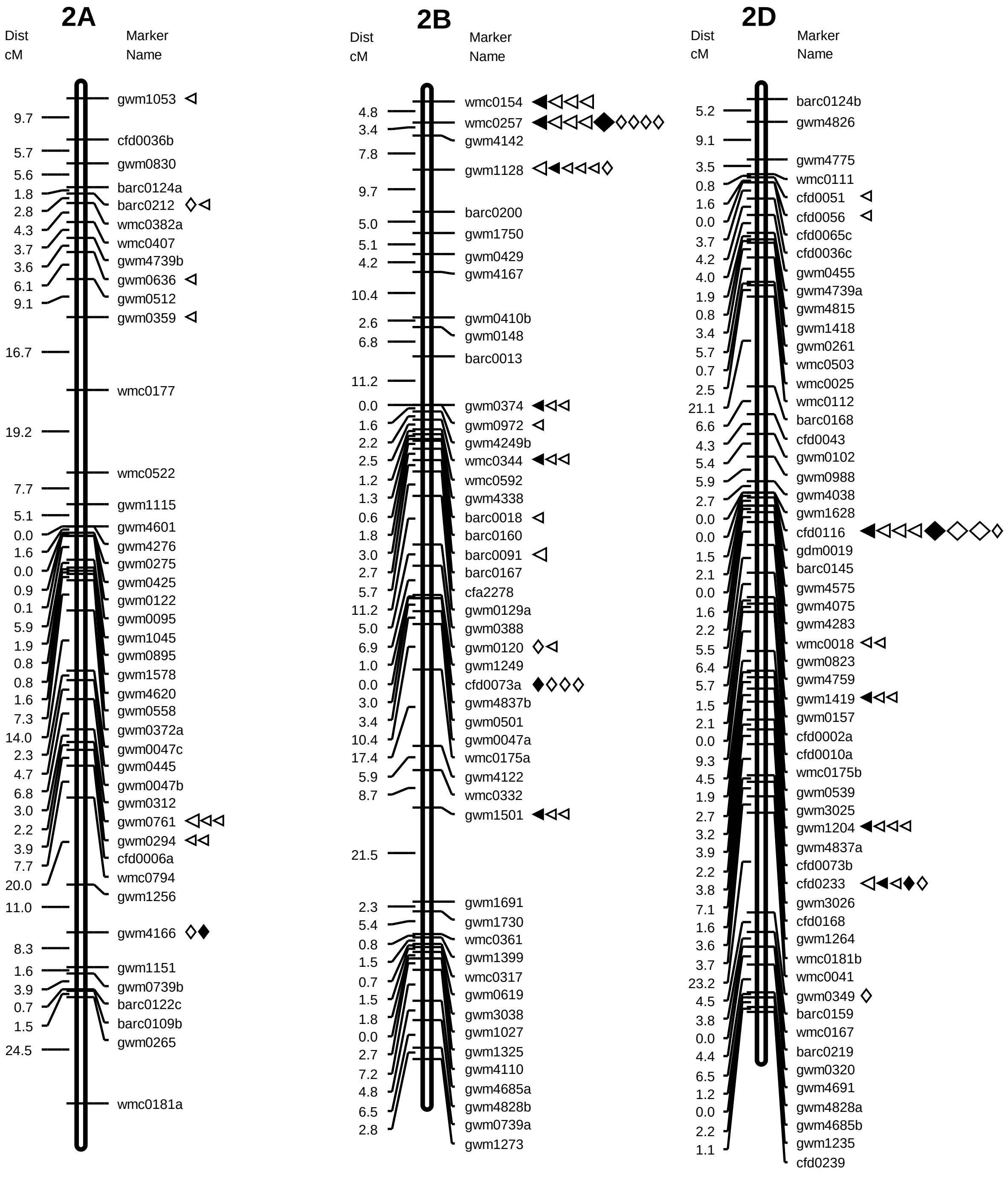

2A
2B
2D
Dist cM
Marker Name

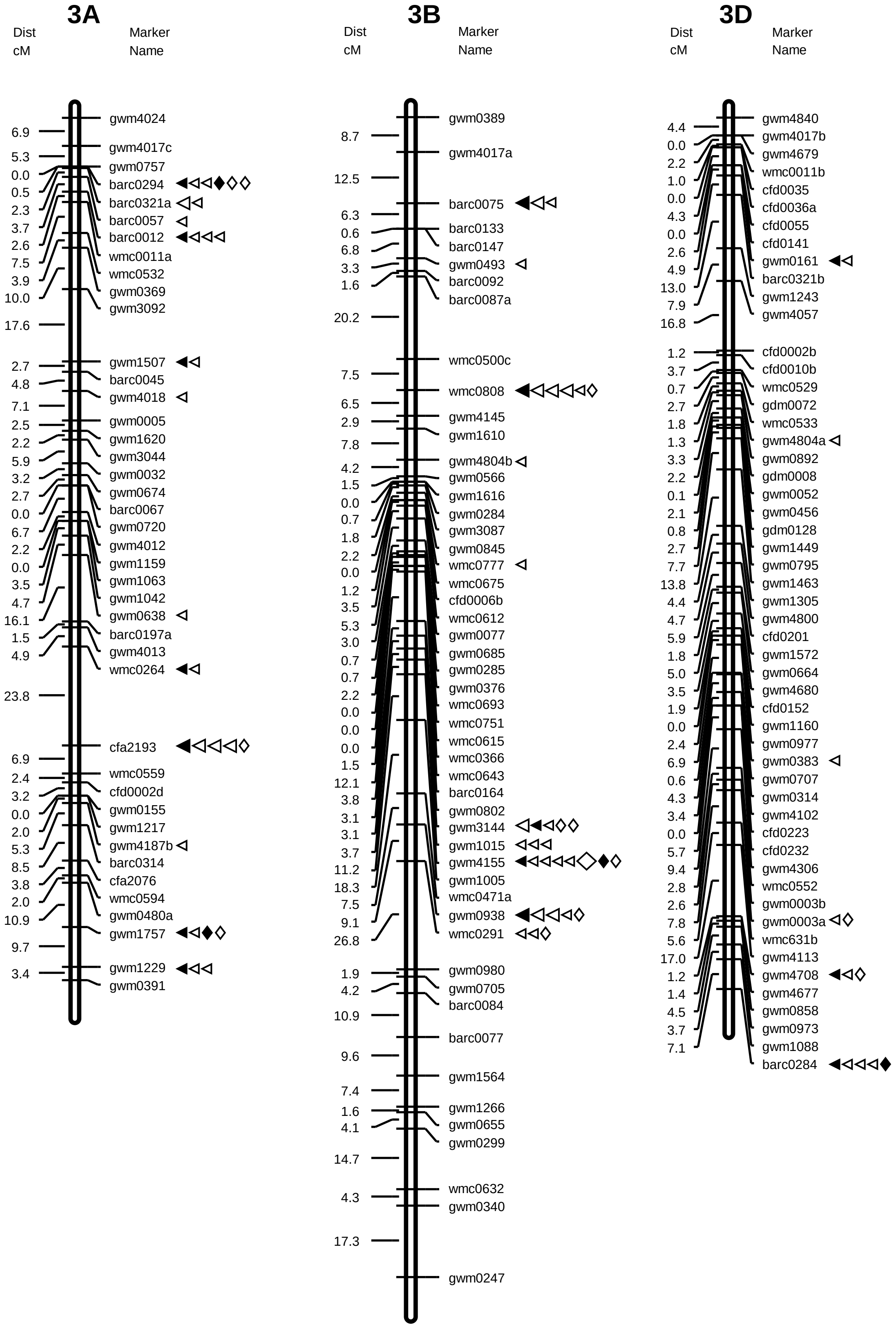
3A
Dist cM
Marker Name
6.9
5.3
0.0
0.5
2.3
3.7
2.6
7.5
3.9
10.0
17.6
2.7
4.8
7.1
2.5
2.2
5.9
3.2
2.7
0.0
6.7
2.2
0.0
3.5
4.7
16.1
1.5
4.9
23.8
6.9
2.4
3.2
0.0
2.0
5.3
8.5
3.8
2.0
10.9
9.7
3.4
gwm4024
gwm4017c
gwm0757
barc0294
barc0321a
barc0057
barc0012
wmc0011a
wmc0532
gwm0369
gwm3092
gwm1507
barc0045
gwm4018
gwm0005
gwm1620
gwm3044
gwm0032
gwm0674
barc0067
gwm0720
gwm4012
gwm1159
gwm1063
gwm1042
gwm0638
barc0197a
gwm4013
wmc0264
cfa2193
wmc0559
cfd0002d
gwm0155
gwm1217
gwm4187b
barc0314
cfa2076
wmc0594
gwm0480a
gwm1757
gwm1229
gwm0391
3B
Dist cM
Marker Name
8.7
12.5
6.3
0.6
6.8
3.3
1.6
20.2
7.5
6.5
2.9
7.8
4.2
1.5
0.0
0.7
1.8
2.2
0.0
1.2
3.5
5.3
3.0
0.7
0.7
2.2
0.0
0.0
0.0
1.5
12.1
3.8
3.1
3.1
3.7
11.2
18.3
7.5
9.1
26.8
1.9
4.2
10.9
9.6
7.4
1.6
4.1
14.7
4.3
17.3
gwm0389
gwm4017a
barc0075
barc0133
barc0147
gwm0493
barc0092
barc0087a
wmc0500c
wmc0808
gwm4145
gwm1610
gwm4804b
gwm0566
gwm1616
gwm0284
gwm3087
gwm0845
wmc0777
wmc0675
cfd0006b
wmc0612
gwm0077
gwm0685
gwm0285
gwm0376
wmc0693
wmc0751
wmc0615
wmc0366
wmc0643
barc0164
gwm0802
gwm3144
gwm1015
gwm4155
gwm1005
wmc0471a
gwm0938
wmc0291
gwm0980
gwm0705
barc0084
barc0077
gwm1564
gwm1266
gwm0655
gwm0299
wmc0632
gwm0340
gwm0247
3D
Dist cM
Marker Name
4.4
0.0
2.2
1.0
0.0
4.3
0.0
2.6
4.9
13.0
7.9
16.8
1.2
3.7
0.7
2.7
1.8
1.3
3.3
2.2
0.1
2.1
0.8
2.7
7.7
13.8
4.4
4.7
5.9
1.8
5.0
3.5
1.9
0.0
2.4
6.9
0.6
4.3
3.4
0.0
5.7
9.4
2.8
2.6
7.8
5.6
17.0
1.2
1.4
4.5
3.7
7.1
gwm4840
gwm4017b
gwm4679
wmc0011b
cfd0035
cfd0036a
cfd0055
cfd0141
gwm0161
barc0321b
gwm1243
gwm4057
cfd0002b
cfd0010b
wmc0529
gdm0072
wmc0533
gwm4804a
gwm0892
gdm0008
gwm0052
gwm0456
gdm0128
gwm1449
gwm0795
gwm1463
gwm1305
gwm4800
cfd0201
gwm1572
gwm0664
gwm4680
cfd0152
gwm1160
gwm0977
gwm0383
gwm0707
gwm0314
gwm4102
cfd0223
cfd0232
gwm4306
wmc0552
gwm0003b
gwm0003a
wmc631b
gwm4113
gwm4708
gwm4677
gwm0858
gwm0973
gwm1088
barc0284

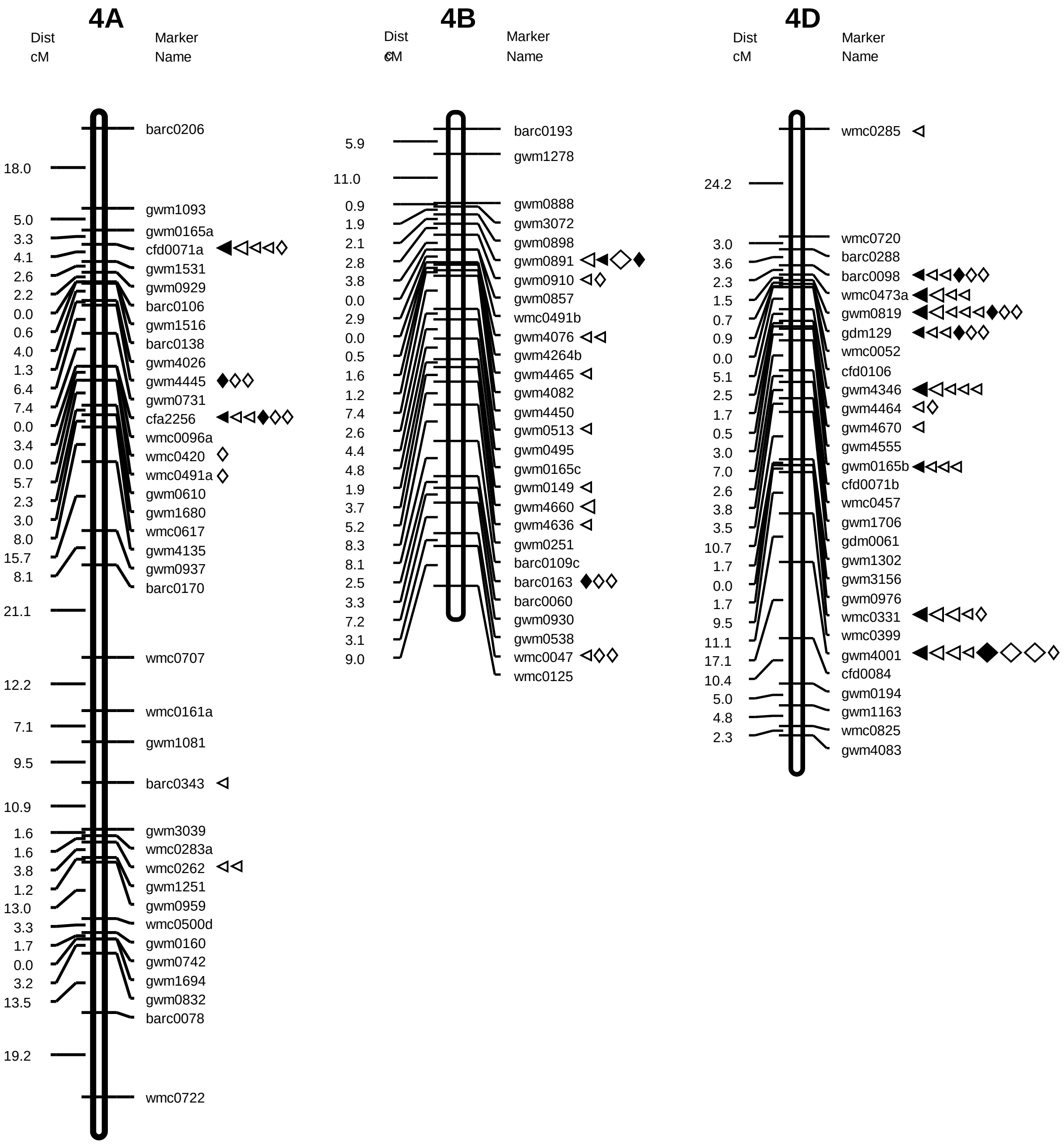

4A
Dist cM
Marker Name
barc0206
18.0
5.0
gwm1093
3.3
gwm0165a
4.1
cfd0071a
2.6
gwm1531
2.2
gwm0929
0.0
barc0106
0.6
gwm1516
4.0
barc0138
1.3
gwm4026
6.4
gwm4445
7.4
gwm0731
0.0
cfa2256
3.4
wmc0096a
0.0
wmc0420
5.7
wmc0491a
2.3
gwm0610
3.0
gwm1680
8.0
wmc0617
15.7
gwm4135
8.1
gwm0937
barc0170
21.1
wmc0707
12.2
wmc0161a
7.1
gwm1081
9.5
barc0343
10.9
1.6
gwm3039
1.6
wmc0283a
3.8
wmc0262
1.2
gwm1251
13.0
gwm0959
3.3
wmc0500d
1.7
gwm0160
0.0
gwm0742
3.2
gwm1694
13.5
gwm0832
barc0078
19.2
wmc0722
4B
Dist cM
Marker Name
barc0193
5.9
gwm1278
11.0
0.9
gwm0888
1.9
gwm3072
2.1
gwm0898
2.8
gwm0891
3.8
gwm0910
0.0
gwm0857
2.9
wmc0491b
0.0
gwm4076
0.5
gwm4264b
1.6
gwm4465
1.2
gwm4082
7.4
gwm4450
2.6
gwm0513
4.4
gwm0495
4.8
gwm0165c
1.9
gwm0149
3.7
gwm4660
5.2
gwm4636
8.3
gwm0251
8.1
barc0109c
2.5
barc0163
3.3
barc0060
7.2
gwm0930
3.1
gwm0538
9.0
wmc0047
wmc0125
4D
Dist cM
Marker Name
wmc0285
24.2
3.0
wmc0720
3.6
barc0288
2.3
barc0098
1.5
wmc0473a
0.7
gwm0819
0.9
gdm129
0.0
wmc0052
5.1
cfd0106
2.5
gwm4346
1.7
gwm4464
0.5
gwm4670
3.0
gwm4555
7.0
gwm0165b
2.6
cfd0071b
3.8
wmc0457
3.5
gwm1706
10.7
gdm0061
1.7
gwm1302
0.0
gwm3156
1.7
gwm0976
9.5
wmc0331
11.1
wmc0399
17.1
gwm4001
10.4
cfd0084
5.0
gwm0194
4.8
gwm1163
2.3
wmc0825
gwm4083

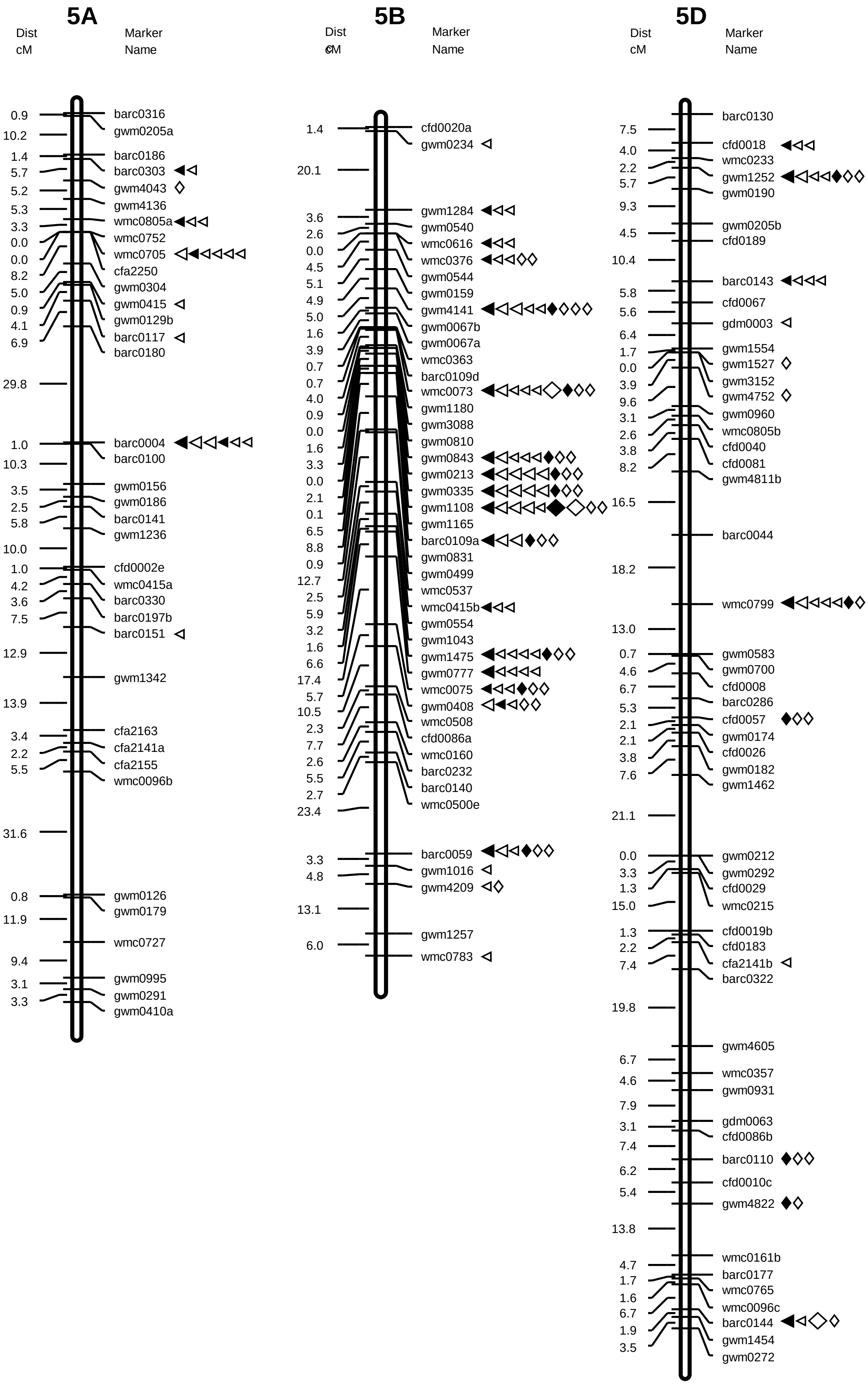
5A
Dist cM
Marker Name
0.9
10.2
1.4
5.7
5.2
5.3
3.3
0.0
0.0
8.2
5.0
0.9
4.1
6.9
29.8
1.0
10.3
3.5
2.5
5.8
10.0
1.0
4.2
3.6
7.5
12.9
13.9
3.4
2.2
5.5
31.6
0.8
11.9
9.4
3.1
3.3
barc0316
gwm0205a
barc0186
barc0303
gwm4043
gwm4136
wmc0805a
wmc0752
wmc0705
cfa2250
gwm0304
gwm0415
gwm0129b
barc0117
barc0180
barc0004
barc0100
gwm0156
gwm0186
barc0141
gwm1236
cfd0002e
wmc0415a
barc0330
barc0197b
barc0151
gwm1342
cfa2163
cfa2141a
cfa2155
wmc0096b
gwm0126
gwm0179
wmc0727
gwm0995
gwm0291
gwm0410a
5B
Dist cM
Marker Name
1.4
20.1
3.6
2.6
0.0
4.5
5.1
4.9
5.0
1.6
3.9
0.7
0.7
4.0
0.9
0.0
1.6
3.3
0.0
2.1
0.1
6.5
8.8
0.9
12.7
2.5
5.9
3.2
1.6
6.6
17.4
5.7
10.5
2.3
7.7
2.6
5.5
2.7
23.4
3.3
4.8
13.1
6.0
cfd0020a
gwm0234
gwm1284
gwm0540
wmc0616
wmc0376
gwm0544
gwm0159
gwm4141
gwm0067b
gwm0067a
wmc0363
barc0109d
wmc0073
gwm1180
gwm3088
gwm0810
gwm0843
gwm0213
gwm0335
gwm1108
gwm1165
barc0109a
gwm0831
gwm0499
wmc0537
wmc0415b
gwm0554
gwm1043
gwm1475
gwm0777
wmc0075
gwm0408
wmc0508
cfd0086a
wmc0160
barc0232
barc0140
wmc0500e
barc0059
gwm1016
gwm4209
gwm1257
wmc0783
5D
Dist cM
Marker Name
7.5
4.0
2.2
5.7
9.3
4.5
10.4
5.8
5.6
6.4
1.7
0.0
3.9
9.6
3.1
2.6
3.8
8.2
16.5
18.2
13.0
0.7
4.6
6.7
5.3
2.1
2.1
3.8
7.6
21.1
0.0
3.3
1.3
15.0
1.3
2.2
7.4
19.8
6.7
4.6
7.9
3.1
7.4
6.2
5.4
13.8
4.7
1.7
1.6
6.7
1.9
3.5
barc0130
cfd0018
wmc0233
gwm1252
gwm0190
gwm0205b
cfd0189
barc0143
cfd0067
gdm0003
gwm1554
gwm1527
gwm3152
gwm4752
gwm0960
wmc0805b
cfd0040
cfd0081
gwm4811b
barc0044
wmc0799
gwm0583
gwm0700
cfd0008
barc0286
cfd0057
gwm0174
cfd0026
gwm0182
gwm1462
gwm0212
gwm0292
cfd0029
wmc0215
cfd0019b
cfd0183
cfa2141b
barc0322
gwm4605
wmc0357
gwm0931
gdm0063
cfd0086b
barc0110
cfd0010c
gwm4822
wmc0161b
barc0177
wmc0765
wmc0096c
barc0144
gwm1454
gwm0272

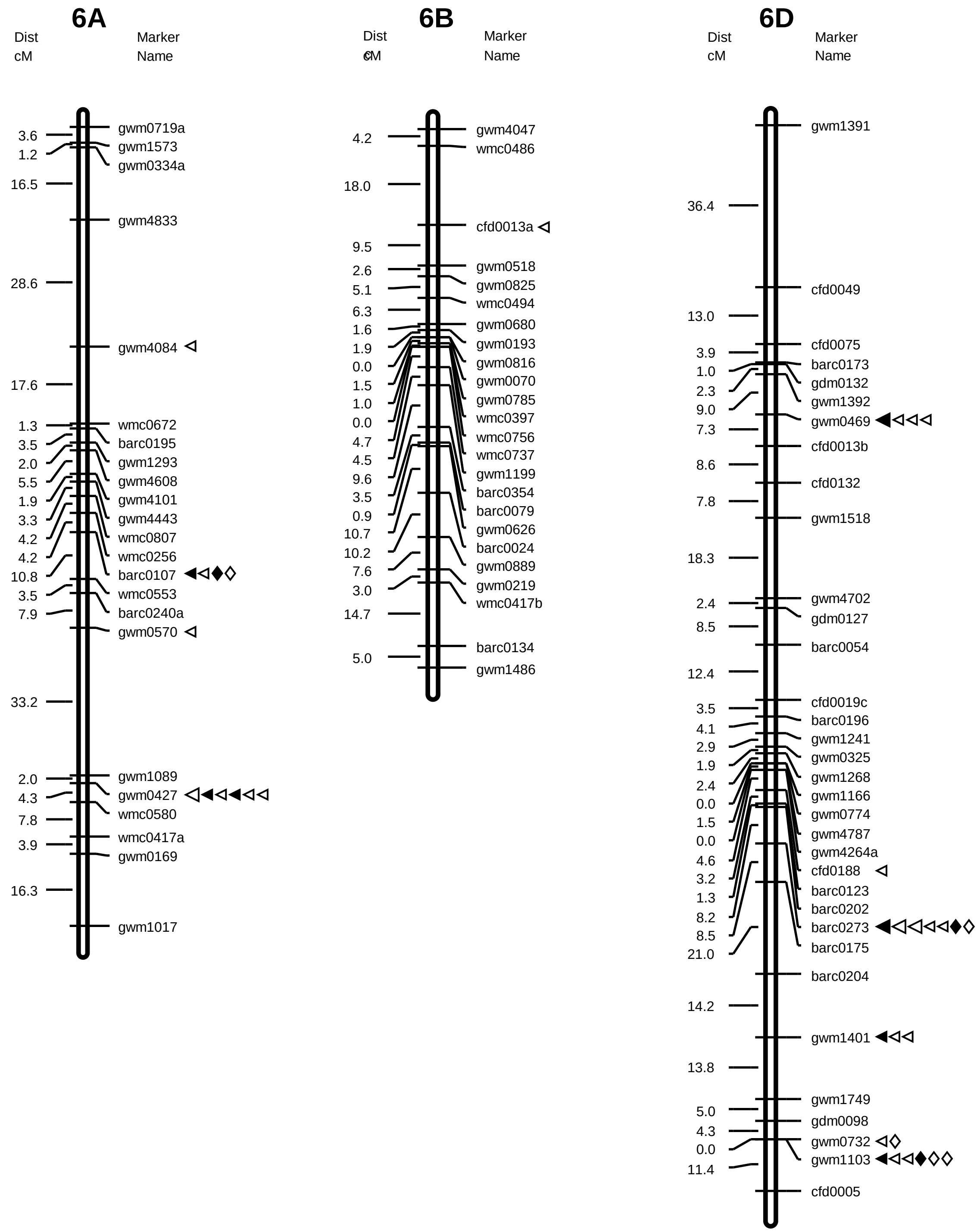
6A
Dist cM
Marker Name
3.6
1.2
16.5
28.6
17.6
1.3
3.5
2.0
5.5
1.9
3.3
4.2
4.2
10.8
3.5
7.9
33.2
2.0
4.3
7.8
3.9
16.3
gwm0719a
gwm1573
gwm0334a
gwm4833
gwm4084
wmc0672
barc0195
gwm1293
gwm4608
gwm4101
gwm4443
wmc0807
wmc0256
barc0107
wmc0553
barc0240a
gwm0570
gwm1089
gwm0427
wmc0580
wmc0417a
gwm0169
gwm1017
6B
Dist cM
Marker Name
4.2
18.0
9.5
2.6
5.1
6.3
1.6
1.9
0.0
1.5
1.0
0.0
4.7
4.5
9.6
3.5
0.9
10.7
10.2
7.6
3.0
14.7
5.0
gwm4047
wmc0486
cfd0013a
gwm0518
gwm0825
wmc0494
gwm0680
gwm0193
gwm0816
gwm0070
gwm0785
wmc0397
wmc0756
wmc0737
gwm1199
barc0354
barc0079
gwm0626
barc0024
gwm0889
gwm0219
wmc0417b
barc0134
gwm1486
6D
Dist cM
Marker Name
36.4
13.0
3.9
1.0
2.3
9.0
7.3
8.6
7.8
18.3
2.4
8.5
12.4
3.5
4.1
2.9
1.9
2.4
0.0
1.5
0.0
4.6
3.2
1.3
8.2
8.5
21.0
14.2
13.8
5.0
4.3
0.0
11.4
gwm1391
cfd0049
cfd0075
barc0173
gdm0132
gwm1392
gwm0469
cfd0013b
cfd0132
gwm1518
gwm4702
gdm0127
barc0054
cfd0019c
barc0196
gwm1241
gwm0325
gwm1268
gwm1166
gwm0774
gwm4787
gwm4264a
cfd0188
barc0123
barc0202
barc0273
barc0175
barc0204
gwm1401
gwm1749
gdm0098
gwm0732
gwm1103
cfd0005

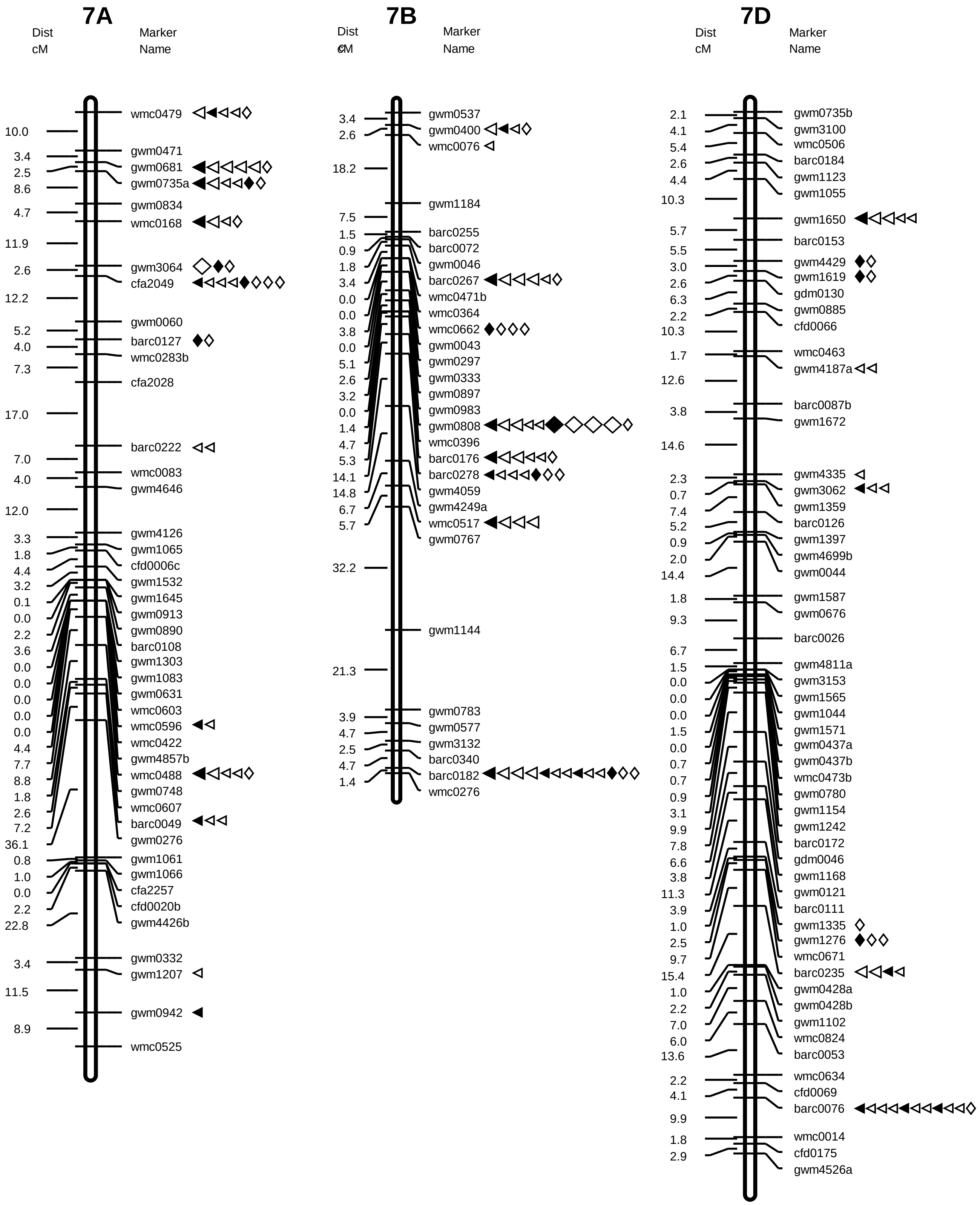
7A
Dist cM
Marker Name
wmc0479
10.0
3.4
gwm0471
2.5
gwm0681
8.6
gwm0735a
4.7
gwm0834
wmc0168
11.9
2.6
gwm3064
cfa2049
12.2
5.2
gwm0060
4.0
barc0127
wmc0283b
7.3
cfa2028
17.0
barc0222
7.0
4.0
wmc0083
gwm4646
12.0
3.3
gwm4126
1.8
gwm1065
4.4
cfd0006c
3.2
gwm1532
0.1
gwm1645
0.0
gwm0913
2.2
gwm0890
3.6
barc0108
0.0
gwm1303
0.0
gwm1083
0.0
gwm0631
0.0
wmc0603
0.0
wmc0596
4.4
wmc0422
7.7
gwm4857b
8.8
wmc0488
1.8
gwm0748
2.6
wmc0607
7.2
barc0049
36.1
gwm0276
0.8
gwm1061
1.0
gwm1066
0.0
cfa2257
2.2
cfd0020b
22.8
gwm4426b
3.4
gwm0332
gwm1207
11.5
gwm0942
8.9
wmc0525
7B
Dist cM
Marker Name
3.4
gwm0537
2.6
gwm0400
wmc0076
18.2
gwm1184
7.5
1.5
barc0255
0.9
barc0072
1.8
gwm0046
3.4
barc0267
0.0
wmc0471b
0.0
wmc0364
3.8
wmc0662
0.0
gwm0043
5.1
gwm0297
2.6
gwm0333
3.2
gwm0897
0.0
gwm0983
1.4
gwm0808
4.7
wmc0396
5.3
barc0176
14.1
barc0278
14.8
gwm4059
6.7
gwm4249a
5.7
wmc0517
gwm0767
32.2
gwm1144
21.3
3.9
gwm0783
4.7
gwm0577
2.5
gwm3132
4.7
barc0340
1.4
barc0182
wmc0276
7D
Dist cM
Marker Name
2.1
gwm0735b
4.1
gwm3100
5.4
wmc0506
2.6
barc0184
4.4
gwm1123
gwm1055
10.3
gwm1650
5.7
barc0153
5.5
3.0
gwm4429
2.6
gwm1619
6.3
gdm0130
2.2
gwm0885
10.3
cfd0066
1.7
wmc0463
gwm4187a
12.6
barc0087b
3.8
gwm1672
14.6
2.3
gwm4335
0.7
gwm3062
7.4
gwm1359
5.2
barc0126
0.9
gwm1397
2.0
gwm4699b
14.4
gwm0044
1.8
gwm1587
9.3
gwm0676
barc0026
6.7
1.5
gwm4811a
0.0
gwm3153
0.0
gwm1565
0.0
gwm1044
1.5
gwm1571
0.0
gwm0437a
0.7
gwm0437b
0.7
wmc0473b
0.9
gwm0780
3.1
gwm1154
9.9
gwm1242
7.8
barc0172
6.6
gdm0046
3.8
gwm1168
11.3
gwm0121
3.9
barc0111
1.0
gwm1335
2.5
gwm1276
9.7
wmc0671
15.4
barc0235
1.0
gwm0428a
2.2
gwm0428b
7.0
gwm1102
6.0
wmc0824
13.6
barc0053
2.2
wmc0634
4.1
cfd0069
barc0076
9.9
1.8
wmc0014
2.9
cfd0175
gwm4526a
