# Supplementary figures and images for: Whole Genome Association Mapping of Fusarium Head Blight Resistance in European Winter Wheat (Triticum aestivum L.)
Source: PLoS One. 2013 Feb 22;8(2):e57500. doi: 10.1371/journal.pone.0057500 (PMC3579808; doi:10.1371/journal.pone.0057500)

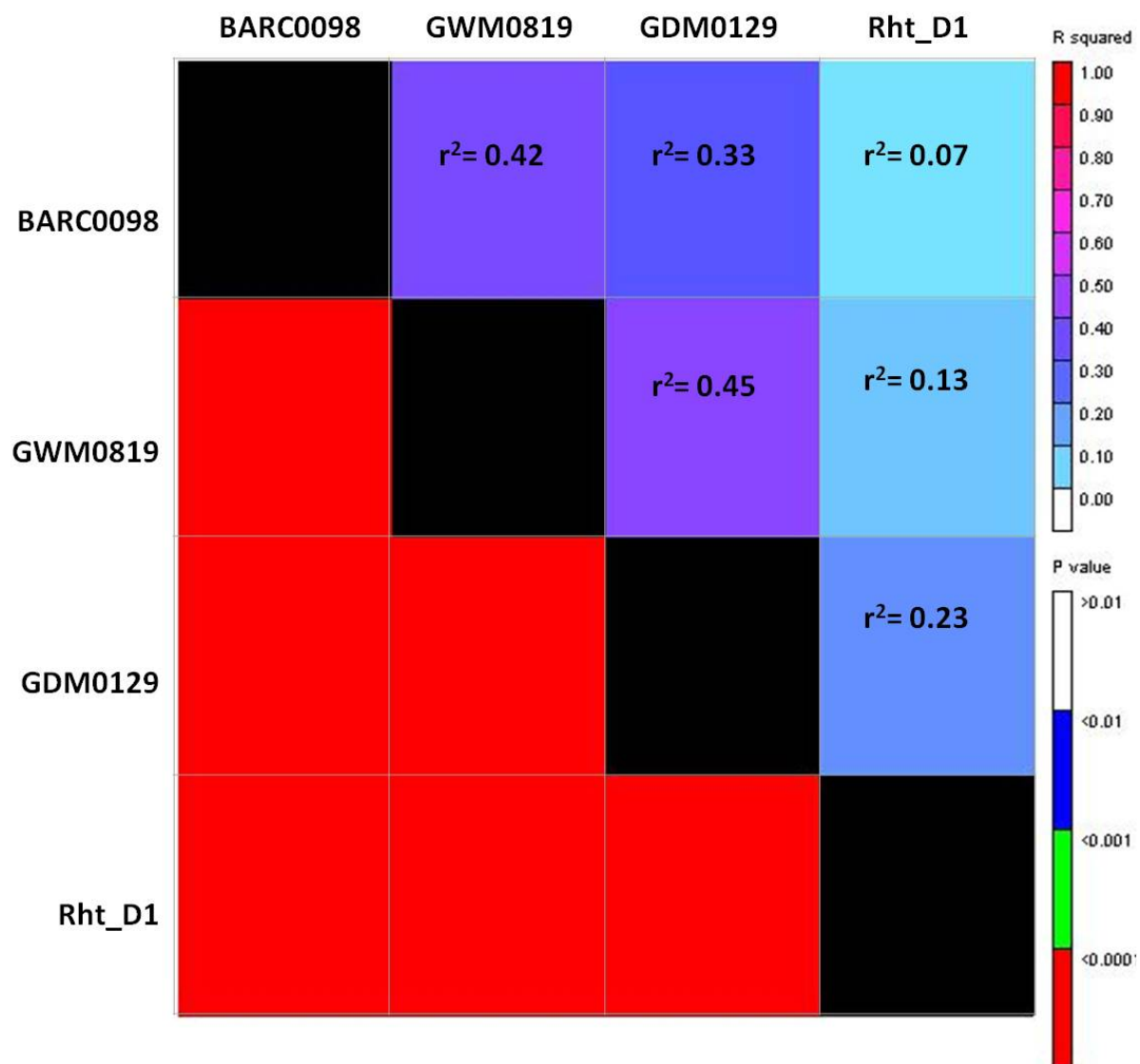

Figure S2: Analysis of linkage disequilibrium (LD) in the region of *Rht-D1* on chromosome 4D.

Supplement: Figure S2 — Analysis of linkage disequilibrium (LD) in the region of Rht-D1 on chromosome 4D. (PDF) [file pone.0057500.s002.pdf]
