# Supplementary material for: Whole Genome Association Mapping of Fusarium Head Blight Resistance in European Winter Wheat (Triticum aestivum L.)
Source: PLoS One. 2013 Feb 22;8(2):e57500. doi: 10.1371/journal.pone.0057500 (PMC3579808; doi:10.1371/journal.pone.0057500)
Supplement: Table S2 — Spearman rank order correlations of FHB score of 372 varieties among four environments and BLUEs. *** P<0.001. (DOCX) [file pone.0057500.s004.docx]

**Table S2: Spearman rank order correlations of FHB scores in 372 varieties among four environments and the BLUEs values.** *** P < 0.001

|  | 2009.AHL | 2010.AHL | 2010.BOD | BLUEs |
| --- | --- | --- | --- | --- |
| 2009.CEC | 0.854*** | 0.584*** | 0.748*** | 0.887*** |
| 2009.AHL |  | 0.634*** | 0.822*** | 0.925*** |
| 2010.AHL |  |  | 0.747*** | 0.823*** |
| 2010.BOD |  |  |  | 0.925*** |
