# Supplementary material for: Whole Genome Association Mapping of Fusarium Head Blight Resistance in European Winter Wheat (Triticum aestivum L.)
Source: PLoS One. 2013 Feb 22;8(2):e57500. doi: 10.1371/journal.pone.0057500 (PMC3579808; doi:10.1371/journal.pone.0057500)
Supplement: Table S3 — Analysis of variance of FHB score in 372 varieties in four environments. (DOCX) [file pone.0057500.s005.docx]

**Table S3: Analysis of variance of FHB score in 372 varieties in four environments.**

|  | DF | SS | MS | F | P |
| --- | --- | --- | --- | --- | --- |
| Genotype | 371 | 65312 | 176 | 9.9 | < 0.001 |
| Environment | 3 | 1962 | 654 | 36.8 | < 0.001 |
| Residual | 1113 | 19800 | 18 |  |  |
| Total | 1487 | 87074 | 59 |  |  |

DF = Degrees of freedom

SS = Sum of Squares

MS = Mean of Squares
