# Supplementary material for: Whole Genome Association Mapping of Fusarium Head Blight Resistance in European Winter Wheat (Triticum aestivum L.)
Source: PLoS One. 2013 Feb 22;8(2):e57500. doi: 10.1371/journal.pone.0057500 (PMC3579808; doi:10.1371/journal.pone.0057500)
Supplement: Table S4 — Spearman rank order correlations of FHB-BLUEs of 372 varieties with HD-BLUEs (heading date) and PH-BLUEs (plant height). P<0.001. (DOCX) [file pone.0057500.s006.docx]

**Table S4: Spearman rank order correlations of FHB-BLUEs of 372 varieties with HD-BLUEs (heading date) and PH-BLUEs (plant height).** P < 0.001

|  | HD-BLUEs | PH-BLUEs |
| --- | --- | --- |
| FHB-BLUEs | - 0.22 | - 0.35 |
